# Very late-onset behavioral variant frontotemporal dementia

**DOI:** 10.1590/S1980-57642013DN70100020

**Published:** 2013

**Authors:** Henrique Cerqueira Guimarães, Tatiana de Carvalho Espindola

**Affiliations:** 1 Geriatric Medicine Residency Program, Hospital Odilon Behrens/Prefeitura Municipal de Belo Horizonte, Belo Horizonte MG, Brazil. Hospital Universitário Risoleta Tolentino Neves, Universidade Federal de Minas Gerais, Belo Horizonte MG, Brazil.

**Keywords:** frontotemporal dementia, very late-onset, dementia, diagnosis

## Abstract

Current concepts regarding frontotemporal lobar degeneration (FTLD) have evolved rapidly in recent years. Genetically determined FTLD cohorts have broadened our knowledge pertaining to its clinical presentation, neuroimaging findings and demographics. In this study we present a case report of a patient diagnosed with behavioral variant frontotemporal dementia diagnosis in her nineties during hospital admission for a ground-level fall. We believe this case reinforces the pervasive nature of this clinical entity, and may contribute to an increased awareness of this diagnostic possibility in late-onset dementia.

## Abstract (pt)

Os conceitos atuais à respeito da degeneração lobar frontotemporal (DLFT) evoluíram rapidamente nos últimos anos. Coortes de pacientes com DLFT geneticamente determinada ampliaram nossos conhecimentos a cerca dos aspectos clínicos, de neuroimagem e demográficos relacionados a esta doença. Aqui nós reportamos o caso de uma paciente que recebeu o diagnóstico de demência frontotemporal em sua variante comportamental aos noventa anos de idade, por ocasião de uma internação hospitalar devido à queda da própria altura. Este caso reforça a ubiquidade desta entidade clínica, e pode contribuir para a conscientização quanto a esta possibilidade diagnóstica em demências de início tardio.

## INTRODUCTION

The frontotemporal lobar degenerations (FTLD) constitute a heterogeneous group of diseases, with multiple and often overlapping clinical phenotypes that may share the same pathological and even genetic underpinnings (for a review see).^1^ The behavioral variant frontotemporal dementia (bvFTD) is the most common presenting syndrome from this group of disorders.^2^ Overall, since the proposal of the first consensus diagnostic criteria in 1994,3 clinicians and researchers were taught that FTD was prototypically a presenile onset dementia associated with profound behavioral disorders, personality changes and relative preservation of episodic memory.

Multicentric cohorts of patients with FTLD consistently point to an average age at onset of around the sixth decade of life.^4^ Recent advances in the genetic and pathological characterization of these patients have been changing the way we view this complex disease. In recent years, several important concepts have been recognized: the involvement of parietal cortex in a significant proportion of patients;^5^ the possibility of severe amnestic presentation from the early stages of the disease;^6^ and also the wide range of ages at onset, as well as the unexpectedly long survival observed in a few patients from genetically determined cohorts,^7^ especially in those carrying mutations in the progranulin gene.^8^

In accordance with this emerging scenario, we present a case report of a female patient with sound neuroimaging findings who received the diagnosis of probable bvFTD in her nineties during a hospital admission for a ground-level fall. Written informed consent was obtained from the patient's family.

**Evaluation at admission and hospital outcome.** A 90-year-old frail^9^ female widow was admitted to the emergency department of a teaching hospital after a fall that resulted in a left femoral neck fracture. Her past medical history was unremarkable, except for well-controlled systemic hypertension, and chronic use of calcium channel blockers for vertigo. Falls had begun occurring only recently. In the two months prior, two other falls had been registered, without major complications.

At first geriatric evaluation the patient had a fluctuating level of consciousness, was inattentive, partially oriented in time and place, and appeared uncomfortable. She was akinetic, hypophonic, and had marked facial hypomimia attributed to drug-induced parkinsonism. A thorough laboratory panel and a brain CT scan were ordered as part of the *delirium* investigation. Brain imaging showed remarkable frontotemporal atrophy without signs of any acute vascular or traumatic injury. Laboratory data disclosed severe kidney failure (creatinine=3.4 mg/dl), with an estimated glomerular filtration rate of less than 15 ml/min, partially compensated metabolic acidosis (pH=7.3; HCO_3_=14 mmol/L), and a suspected urinary tract infection. Intravenous antibiotics and fluids were given, and a thorough functional evaluation was performed by interviewing her daughter (see below).

In spite of the above-mentioned treatments, her clinical condition was complicated by septic shock. The medical staff discussed the available therapeutic alternatives with her family and agreed to adopt a treatment strategy with emphasis on comfort in the ward setting. A multidisciplinary palliative care team provided support for the patient and her family. Despite mild clinical and hemodynamic improvement in the ensuing days, the patient had persistent sleepiness, inattention and disorientation, which precluded cognitive testing. Shortly after, her clinical condition worsened again, because of a probable hospital-acquired pneumonia, and she died within a few days.

**Functional status.** Since the patient's precarious clinical condition precluded any performance-based assessment, all the information below was provided by a daughter and a son-in-law, who lived together with her. No family history of dementia or behavioral disorders was acknowledged by the informants. The patient was still completely independent in basic activities of daily living, with a fully preserved Katz Index.^10^ Personal grooming was satisfactory. Instrumental activities of daily living where progressively abandoned, according to relatives, mostly because of lack of interest. Cognitive problems, especially memory complaints, were not mentioned by her family. No prosopagnosia or significant anomia could be recalled by her family. Temporal orientation was also preserved. Her routine at home was strict and she could manage this without much supervision. No outdoor activities had been undertaken for the last five years. Management of basic finances was abandoned for at least eight years. In the last few years she needed help to take her medicines. Her score on the Functional Activities Questionnaire^11^ was 15 out of 30 possible points, where lower scores indicate mean better functional performance. Her score on the Frontotemporal Dementia Rating Scale (FRS)^12^ was 41%, indicating a late-moderate stage of disease. No sensory or gross mobility impairments could justify her functional dependence. Depressive symptoms were mild and occasional.

## Behavioral assessment

*Apathy - lack of interest* - Cooking and attending church services used to be her preferred activities. She used to cook well on a daily basis until five years earlier, including the preparation of meals for a great number of people in the family. This habit was progressively abandoned. Her daughter thought she was too old to maintain this activity, although could not remember any major mistakes made when cooking in the last few years. At approximately the same period, she stopped attending church services and since then had been praying out loud at home for a brief period every day at the same time.

*Compulsive and ritualistic behaviors* - In the last six years she had developed compulsive water drinking, without any clinical condition justifying this urge. This behavior occurred also at night and caused sleep deprivation. She had her own place on the sofa, where nobody else could sit. The lunch had to be served at noon and bathing occurred at 15h strictly. Every time she went to the kitchen, she stood to face the wall clock and read the current time out loud. Until admission to hospital, she used to cook her evening flummery unaided, made from the same oatmeal brand, and using the same dishes every day. She had also been hoarding junk in her wardrobe for the last two years.

*Eating behavior* - The patient had been consuming an excessive amount of pasta over the last five years, especially instant noodles. She developed the habit of storing candies in her room for the last three years. In recent months she had also been eating from the pans in the kitchen, despite having recently dined.

*Empathic concern and social withdrawal* - There was a suspected empathic symptom ten years before, at the death of the most beloved grandson. Most of the family members thought that her reaction was blunted. After this event, insidiously, nobody else in the family was able to elicit a positive affective reaction from her, except one of the granddaughters. At the same time she developed an unusual preference for a dark ambience. She had no reasonable explanation for this behavior. These symptoms were regarded as depressive, but no treatment was prescribed and no other depression symptom developed. For the last two years she had taken to deliberately leaving the room when visitors arrived, and she had also started playing with dolls.

## DISCUSSION

We reported a case of a nonagenarian woman with progressive functional decline for instrumental activities of daily living and mild behavioral changes dating back at least five years. In the current evaluation she was considered at a late-moderate stage of dementia according to FRS.^12^ Despite multiple behavioral changes and unequivocal functional impairment, these symptoms emerged very insidiously and were regarded by relatives as a natural consequence of the aging process. No medical visit was scheduled to investigate these features and the patient kept up regular visits only to her cardiologist.

There are very few available reports on late onset bvFTD. In a large Swedish community-based survey of late-onset behavioral disorders,^13^ frontal lobe behavioral syndrome was present in 19.1% of the population. Of these, 87% had dementia of either Alzheimer's disease or vascular etiology. bvFTD was supposed to explain the frontal behavioral symptoms in 3.1% (n=14) of the evaluated population. This study however, had several serious limitations: cognitive assessment was not performed; almost half of the supposed bvFTD patients lacked neuroimaging data; and 64% of these patients could not be considered demented according to the selected diagnostic criteria. In two other fairly large European case series in which age at onset was analyzed, patients considered to have late or very late onset disease were, on average, around 70 years old and displayed clinical and pathological findings similar to those of early-onset patients.^14,15^ It is estimated that almost 25% of bvFTD cases have an age at disease onset of over 65 years.^16^ Subjects from large cohorts of progranulin mutation carriers displayed the widest range of ages at onset, with a few subjects whose symptoms started in their eighties.^8,17^ No genetic testing data was available for our patient, and no family history consistent with familial FTD was gathered.

According to caregiver information and current staging it is believed that disease symptoms started some 5 to 6 years earlier. This delay is not unusual in Brazil, and the reasons that underpin this phenomenon are still poorly understood, although cultural issues have been proposed as an explanation.^18^ It remains to be investigated if the above-described behavioral profile, characterized by an organized and stereotyped home routine, without overt disinhibition and memory impairment, could delay family awareness of an underlying neurodegenerative disease. The absence of objective cognitive assessment and neuropathological data constitute important limitations of the present report.

In conclusion, we described an oldest-old patient with behavioral and neuroimaging features unequivocally consistent with probable bvFTD, whose diagnosis was reached by chance after emergency department admission because of a fall, hip fracture and *delirium* onset. We believe that this case reinforces the pervasive nature of this clinical entity, and may contribute to increased awareness of this diagnostic possibility in very late-onset dementia.

## Figures and Tables

**Figure 1:**
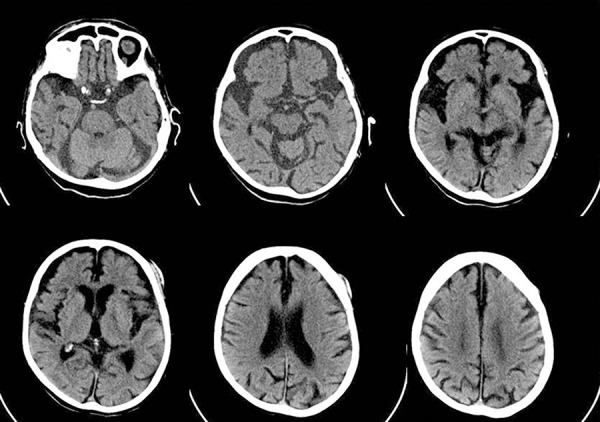
Brain computed tomography scan showing moderately severe atrophy in frontal lobes, especially in ventral portions and bilateral anterior temporal lobes.
